# Atorvastatin Sensitizes Breast and Lung Cancer Cells to Ionizing Radiation

**DOI:** 10.22037/ijpr.2020.15487.13126

**Published:** 2020

**Authors:** Seyed Jalal Hosseinimehr, Fatemeh Ghasemi, Farzaneh Flahatgar, Najmeh Rahmanian, Arash Ghasemi, Hossein Asgarian-Omran

**Affiliations:** a *Department of Radiopharmacy, Faculty of Pharmacy, Mazandaran University of Medical Sciences, Sari, Iran. *; b *Department of Radiology and Radiation Oncology, Faculty of Medicine, Mazandaran University of Medical Sciences, Sari, Iran. *; c *Department of Immunology, School of Medicine, Mazandaran University of Medical Sciences, Sari, Iran. *; d *Immunogenetics Research Center, School of Medicine, Mazandaran University of Medical Sciences, Sari, Iran.*

**Keywords:** Atorvastatin, Radiosensitizing, Apoptosis, Ionizing radiation, Radiosensitizer

## Abstract

Tumour cells may be resistant to radiotherapy that results in unsuccessful cancer treatment in patients. The aim of this study was to evaluate the sensitizing effect of atorvastatin (ATV) on breast cancer (MDA-MB-231) and non-small cell lung cancer (A-549) cells following exposure to ionizing radiation (IR). These cells were treated with ATV and exposed to X-ray at dose 4 Gy. The radiosensitizing effects of ATV were evaluated by flow cytometry and anti-proliferation assays. The production of reactive oxygen species (ROS) was determined in irradiated and treated cells with ATV. The findings of this study showed that ATV increased the percentage of apoptotic cells in irradiated breast and lung cancer cells. ATV exhibited anti-proliferative effect on cancer cells and increased cell death induced by IR. ATV increased ROS production in irradiated cells. The present study demonstrates that ATV has radiosensitizing effect on breast and lung cancer cells through increasing apoptosis, ROS production and cell death induced by IR.

## Introduction

Radiotherapy is one of the most effective strategies for cancer treatment. In this treatment protocol, ionizing radiation (IR) is passing through cells and produces free radical and reactive oxygen species (ROS) in cellular environment. These toxic substances could attack to critical macromolecule such as DNA that cause damage. The extensive DNA damage results in cell death (1). However, IR is causing cell death; cancer cells activate endogenous radioresistance signaling pathways in response to killing effect of IR (2, 3). Radioresistance response in tumor cells is the major challenge in cancer treatment. To make overcoming resistance of the cancer cells to IR, there are several approaches as increasing dose of IR or use of radiosensitizers (4). The use of high dose of IR induces side effects on normal tissue that is leading mismanagement of patient during treatment. The administration of a radioprotective agent reduces side effects induced by IR on normal cells and tissues (5, 6). Radiosensitizer is able to sensitize cancer cells to IR (4). Therefore, blocking the cellular repair using radiosensitizer can enhance the efficacy of cancer treatment (7). Atorvastatin (ATV) is a member of statin family that is inhibiting HMG-CoA reductase activity and is a cholesterol-loweing drug. It exhibits anti-cancer activity in several human cancer cells (8-10). ATV is able to induce autophagy in cancer cells leads to cell death (11, 12). However, ATV has exhibited anti-cancer effects, several studies demonstrated the radioprotective effect of ATV on normal tissues. Recently, we demonstrated that ATV reduced side effects of IR on lymphocyte, testis, and kidney (13-15). Anti-inflammatory, enhanced endogenous antioxidant, inhibition of lipid peroxidation, and caspase-3 are main mechanisms involved in radioprotective effect of ATV on normal cells and tissues. However, the protective effects of ATV on normal cells and tissues have been well studied. There are limited studies about the effect of co-treatment of ATV and IR on cancer cells. In a clinical trial, we demonstrated that topical administration of ATV reduced skin toxicity induced by IR in patients with breast cancer (16). Thus, the present study attempts to investigate the sensitizing effect of ATV on IR-induced apoptosis and cell death in breast cancer (MDA-MB-231) and non-small cell lung cancer (A-549) cells. These results demonstrate ATV is potentially radiation sensitizer for the treatment of human breast and lung cancers.

## Experimental

MDA-MB-231 and A-549 cell lines were obtained from the Pasteur Institute (Iran) and cultured at 37 °C and 5% CO_2_ in Dulbecco’s Modified Eagle Medium (DMEM) (Gibco, Paisley, UK) supplemented with 10% fetal bovine serum (FBS) (Gibco, UK) and 100 μg/mL penicillin–streptomycin (Gibco, UK). Annexin-V-FLUOS Staining Kit was obtained from eBioscienc (USA). Atorvastatin (from Sobhan daru Pharmaceutical Company, Iran) was freshly prepared in DMSO as stock solution and diluted in culture medium at concentration dose 10 µM. This ATV concentration was chosen based on our previous study that ATV exhibited *in-vitro* the highest radioprotective effects on human normal lymphocyte without any cellular toxicity as compared to the other concentrations (13).

*Apoptosis determination by flow cytometry*

Apoptotic cells were analysed in cancer cells at 24 h after exposure of cells to IR. MDA-MB-231 and A-549 cells (3 × 10^5^) were treated with ATV at concentration 10 μM for four hours in 12-well plates and then were exposed to IR at dose 4 Gy. The cells were irradiated with 4 MV X-ray produced by a radiotherapy machine (Linear accelerator, Siemens, Primus, Germany) at a 1.96 Gy/min dose rate and source to sample distance (SSD) of 60 cm. The cells were cultured in DMEM medium for 24 h. The apoptotic cells were determined using an ‘Annexin-V-FLUOS Staining Kit’ according to the manufacturer’s instruction (eBioscience, USA). Briefly, the cells were washed with PBS and incubated with Annexin-V FLUOS labeling solution (containing 2 μL Annexin-V-FLUOS labelling reagent and 2 μL propidium iodide (PI) solution in 100 μL incubation buffer for each sample) at room temperature in the dark for 15 min. Following incubation, the analysis was performed with a Partec flow cytometer system and Flomax software (Partec, Germany). Unstained cells were considered as negative control for background determination. For each sample, a minimum of 10000 events were counted and analysed.

*Antiproliferation assay*

MDA-MB-231 and A-549 cells (10,000 cells) were plated in each well of a 96-well plate and were incubated and allowed to attach for 24 h in a humidified atmosphere of 5% CO_2_ in air at 37 °C (Incubator-Biotek-NB 203L Korea). After incubation, the cells were treated with ATV (10 µM) and incubated for 4 h before exposure to X-ray at dose 4 Gy. The radiation dose was selected according to our preliminary study. The cells were incubated for 72 h in a humidified atmosphere of 5% CO_2_ in air at 37 °C. The culture medium was removed and MTT solution (5 mg/mL PBS) was then added and the plate was located in optimal atmosphere at 37 °C. The metabolically active cells reduced MTT to blue formazan crystals. After incubating for 4 h, the formazan crystals in each well were dissolved in DMSO. The absorbance of each well was read with an ELISA Reader at 570/630 nm (Bioteck, USA). The cells without any treatment were used as control for comparison of absorbance and cell viability (17).

*ROS determination*

The intracellular accumulation of ROS was assayed using DCFH-DA, an uncharged, cell permeate fluorescent probe. Inside the cells, DCFH-DA is cleaved by nonspecific esterases forming DCFH, which is the non-fluorescein form and is oxidized to the fluorescent compound DCF in the presence of ROS. MDA-MB-231 and lung cancer cells were seeded in 96 well-plates at a density of 2 × 10^4 ^cells per well overnight. The cells were treated with ATV (10 μM) for 4 h. Afterwards the cells were washed with PBS and loaded with 30 µM DCFH-DA in PBS for 30 min. DCFH-DA was removed and each well was loaded with PBS. The plate was irradiated with X-ray at a single dose of 4 Gy. Since the ROS oxidize intracellular DCFH to the fluorescent DCF dye, the DCF fluorescence intensity is taken as being directly proportional to the ROS concentration. The intensity of fluorescence was recorded by a fluorescence spectrophotometer, with an excitation filter of 490 nm and an emission filter of 520 nm. The ROS level was calculated as the mean intensity of the treated cells.

*Statistical analysis*

The data values are presented as means ± standard deviation (SD). Statistical analysis was performed using one-way analysis of variance (ANOVA), as well as Tukey’s multiple comparison and Dunnett multiple comparison tests. *P*-value < 0.05 was considered as significant and highly significant (Prism 7 Software, 2016, USA).

## Results

*Apoptosis in MDA-MB-231 cells treated with ATV and radiation*

**Figure 1:**
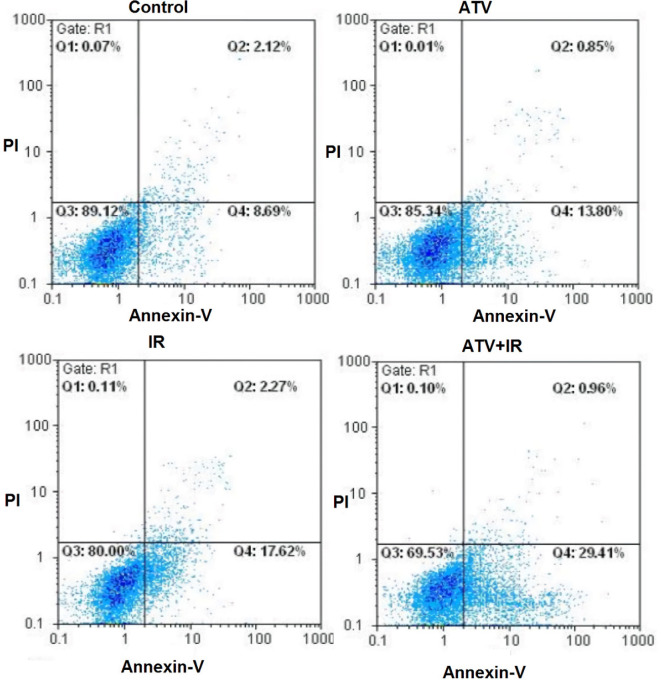
Effect of atorvastatin (ATV; 10 µM) on X-ray-induced apoptosis in MDA-MB-231 cells. Cells were analyzed for Annexin V binding and for PI uptake using flow cytometry. Representative dot plots of one set of five independent experiments of Annexin V and PI staining are shown. In each figure, the lower left quadrant (Annexin V^−^ and PI^−^) was considered as live cells, the lower right quadrant (Annexin V^+^ and PI^−^) was considered as early apoptotic cells, the upper right quadrant (Annexin V^+^ and PI^+^) was considered as late apoptotic and necrotic cells

The flow cytometry was used for measurement of apoptosis in breast cancer cells which were treated with ATV and exposed to IR (Figure 1).

**Figure 2:**
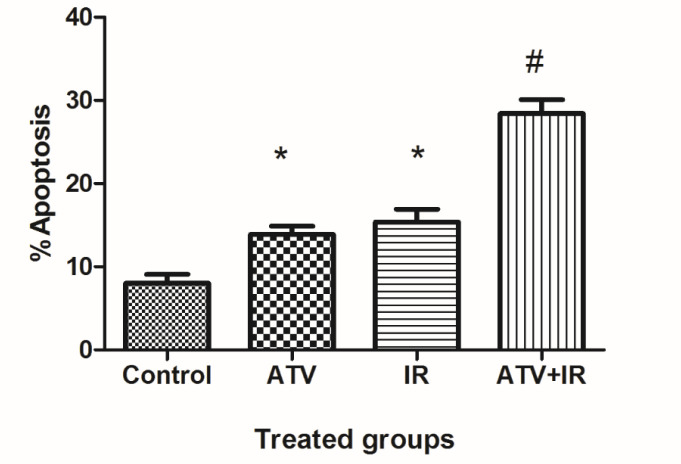
Effect of atorvastatin (ATV; 10 µM) on X-ray ionizing radiation (IR)-induced apoptosis in MDA-MB-231 cells. The percentages of apoptotic cells were shown in experimental groups. Values are expressed as mean ± SD of three independent experiments. ^*^P < 0.05: ATV or 4 Gy group alone compared to sham control, ^#^ATV+ IR and ATV and IR alone groups

As shown in Figure 2, compared to the control group (8.0% ± 2.1), the apoptosis rate of MDA-MB-231 cells was increased in ATV-alone group (13.9% ± 2) and IR-alone group (15.4% ± 3.1) (*P* < 0.01). A non-significant difference was observed between ATV and IR groups in induction of apoptosis in MDA-MB-231 cells was observed. Furthermore, the apoptosis rate was significantly increased in MDA-MB-231 + IR group (28.4% ± 3.3) compared to the IR alone and ATV alone groups (*P* < 0.001).

*Apoptosis in A-549 cells treated with ATV and radiation*

**Figure 3:**
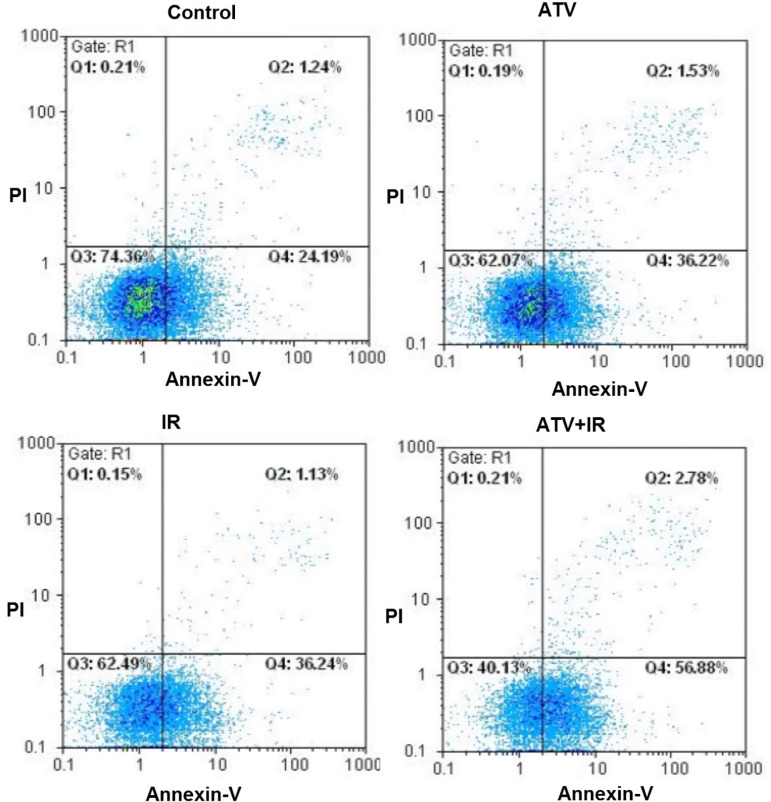
Effect of atorvastatin (ATV; 10 µM) on X-ray radiation-induced apoptosis in A-549 cells. Cells were analyzed for Annexin V binding and for PI uptake using flow cytometry. Representative dot plots of one set of five independent experiments of Annexin V and PI staining are shown. In each figure, the lower left quadrant (Annexin V^−^ and PI^−^) was considered as live cells, the lower right quadrant (Annexin V^+^ and PI^−^) was considered as early apoptotic cells, the upper right quadrant (Annexin V^+^ and PI^+^) was considered as late apoptotic and necrotic cells

**Figure 4:**
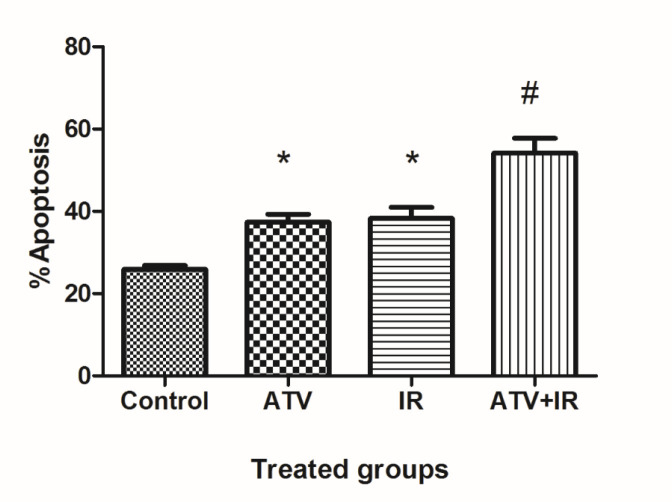
Effect of atorvastatin (ATV; 10 µM) on X-ray ionizing radiation (IR)-induced apoptosis in A-549 cells. The percentages of apoptotic cells were shown in experimental groups. Values are expressed as mean ± SD of three independents experiments. ^*^P < 0.05: ATV or 4 Gy group alone compared to sham control, ^#^ATV+ 4 Gy and ATV and IR alone groups

The flow cytometry was used for measurement of apoptosis in lung cancer cells which were treated with ATV and exposed to IR (Figure 3). As shown in Figure 4, compared to the control group (25.9% ± 1.9), the apoptosis rate of A-549 cells was increased in ATV-alone group (37.6% ± 3.9) and IR-alone group (38.3% ± 4.5) (*P *< 0.05). A non-significant difference was observed between ATV and IR groups in induction of apoptosis in A-549 cells was observed. Furthermore, the apoptosis rate was significantly increased in A-549 + IR group (54.2% ± 7.2) compared to the IR alone and ATV alone groups (*P* < 0.01).

*Effect of ATV and ionizing radiation on cell proliferation in MDA-MB-231*

**Figure 5:**
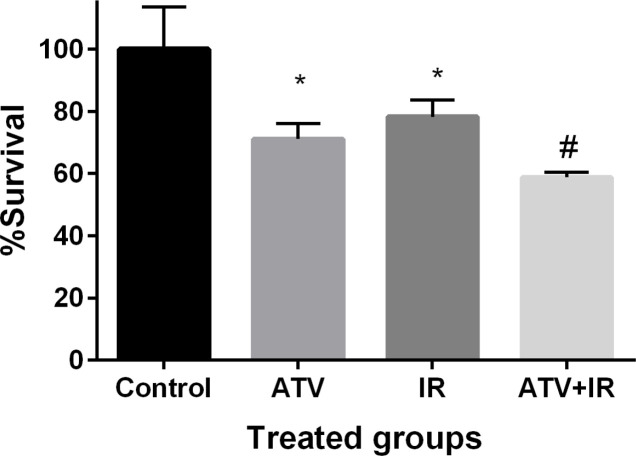
Effect of atorvastatin (ATV) at concentration 10 µM alone and in combination with X-ray ionizing radiation (IR) on MDA-MB-231 cells. Cell proliferation was assayed with MTT test after 72 h incubation. ^*^P < 0.05, comparison control group with IR or ATV alone groups; ^#^*P* < 0.05, comparison ATV + IR groups with IR or ATV alone groups

The effect of ATV on proliferation of breast cancer cell was determined by MTT assay. Breast cancer cells proliferation was significantly inhibited by ATV at 10 µM concentration after 72 h incubation (*P* < 0.01) (Figure 5). ATV exhibited a survival rate of 71% ± 5 in MDA-MB-231 cells. IR exhibited a survival rate of 78% ± 5 in MDA-MB-231 cells at dose 4 Gy (*P* < 0.01). The proliferation of breast cancer cells was significantly reduced by ATV in combination with IR. This additive effect was 59% ± 1 in proliferation of breast cancer cells. A significant difference was observed between ATV+ IR and ATV or IR alone (*P* < 0.05) was observed. This result indicates that ATV has additive effect with X-ray on inhibition of cell growth in breast cancer cell. A radiosensitizing effect of ATV on the breast cancer cells was observed.

*Effect of ATV and ionizing radiation on cell proliferation in A-549 cells*

**Figure 6:**
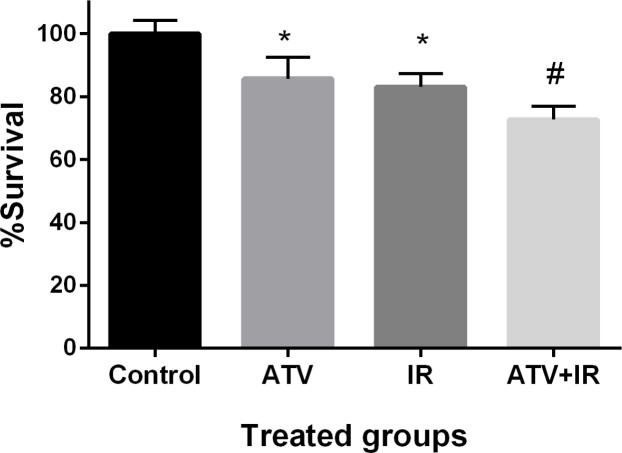
Effect of atorvastatin (ATV) at concentration 10 µM alone and in combination with X-ray ionizing radiation (IR) on A-549 cells. Cell proliferation was assayed with MTT test after 48 h incubation. ^*^P < 0.05, comparison control group with IR or ATV alone groups; ^#^*P* < 0.05, comparison ATV + IR groups with IR or ATV alone groups

The effect of ATV on proliferation of lung cancer cell was determined by MTT assay. Non-small cell lung cancer cells proliferation was significantly inhibited by ATV at 10 µM concentration after 72 h incubation (*P* < 0.01) (Figure 6). ATV exhibited a survival rate of 85% ± 7 in A-549 cells. IR exhibited a survival rate of 83% ± 4 in A-549 cells at dose 4 Gy (*P* < 0.01). The proliferation of lung cancer cells was significantly reduced by ATV in combination with IR. This additive effect was 73% ± 4 in proliferation of lung cancer cells. A non-significant difference was observed between ATV+IR and ATV or IR alone (*P* < 0.05) was observed. This result indicates that ATV has additive effect with X-ray on inhibition of cell growth in the lung cancer cell. A radiosensitizing effect of ATV on the lung cancer cells was observed. 

*ROS levels*

**Figure 7:**
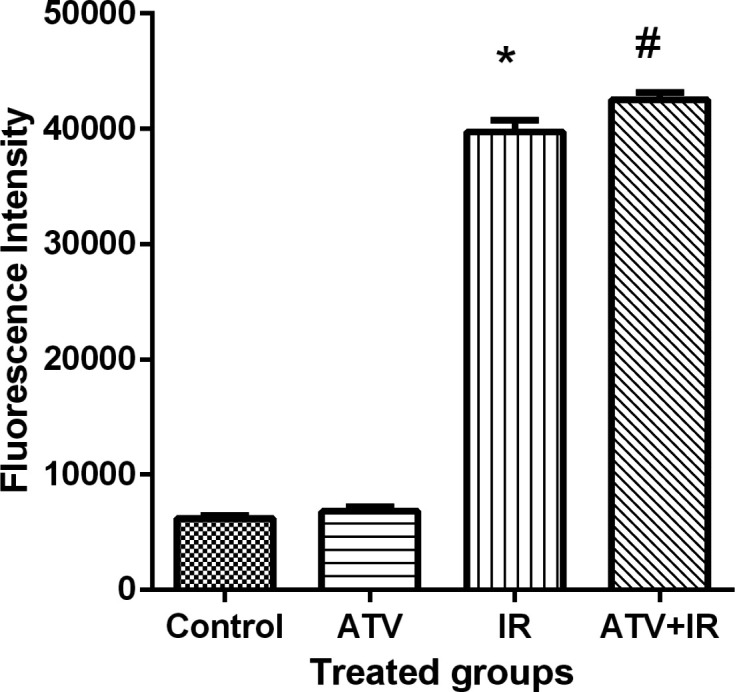
Effect of atorvastatin (ATV) at concentration 10 µM alone and in combination with X-ray ionizing radiation (IR) on reactive oxygen species (ROS) production in MDA-MB-231 cells. ROS were determined with DCF fluorescence assay. ^*^P < 0.001, comparison between IR group with control and ATV groups; ^#^*P* < 0.05 comparison between ATV + IR with IR

**Figure 8:**
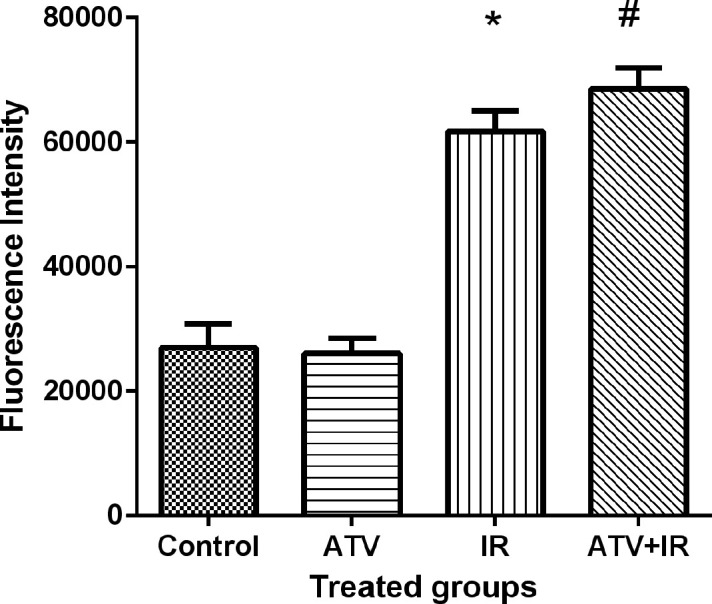
Effect of atorvastatin (ATV) at concentration 10 µM alone and in combination with X-ray ionizing radiation (IR) on reactive oxygen species (ROS) production in A-549 cells. ROS were determined with DCF fluorescence assay. ^*^P < 0.001, comparison between IR group with control and ATV groups; ^#^*P* < 0.05 comparison between ATV+ IR with IR

Ionizing radiation produces ROS and oxidative stress result in cellular toxicity. Irradiated MDA-MB-231 and A-549 cells showed a significant increase in DCF fluorescence after 4 Gy irradiation (*P* < 0.001) (Figure 7). Treatment cells with 10 µM ATV significantly increased ROS levels in irradiated cells (*P* < 0.05) (Figure 8). Thus, pre-treatment with ATV showed an additive effect on radiation-induced ROS generation in the irradiated breast and lung cancer cells**.**

## Discussion

In this study we demonstrated that ATV sensitized MDA-MB-231 breast and A-549 lung cancer cells to IR. ATV increased apoptosis and cell deaths induced by IR on the breast and lung cancer cells. The resistance of the cancer cells to IR is a challenge in radiotherapy regimen in the patients. The cancer cells activate various signalling pathways resulting in resistance to radiation-induced cell death (3). In cancer treatment, it is very benefit to find a medication which enhances killing effect of IR on cancer cells. The administration of a safe radiosensitizer by the patient during radiotherapy results in the improved cancer treatment, also dose of radiation for cancer treatment could be reduced in the patients (3, 4). It is noticed that a radiosensitizer should be ineffective or has radioprotective effect on the normal cells, otherwise radiosensitizer enhances radiation-induced toxicity on the normal cells. ATV is widely used as a medicine for lowering cholesterol in the patients. Recently we showed ATV protected normal human lymphocytes to IR. ATV treatment reduced genotoxicity induced by IR on lymphocytes that micronuclei assay was performed for evaluation of its radioprotective effect. The maximum radioprotection was observed with *in-vitro* ATV treatment at dose 10 µM on irradiated normal human lymphocytes with an efficacy of 68% protection. Genotoxicity was not observed with ATV alone at concentration 10 µM on normal human lymphocytes (13). It is interesting that ATV exhibited a radiosensitizing effect on the breast and lung cancer cells at concentration of 10 µM while ATV exhibited radioprotective effect on normal cells at this dose. IR causes free radicals in cellular environment that these toxic substances have crucial role in cell death. The dose 4 Gy of IR was chosen based on previous studies that this dose was able to increase apoptosis in cancer cells (18-20). In this study, we showed that exposure of cancer cells with IR at dose 4 Gy significantly increased ROS production, and an additive ROS production was observed in cancer cell pretreated with ATV. Findings showed that enhanced ROS production is a mechanism involved in cancer cell deaths that were exposed to IR. Previous studies showed that ATV has anticancer effect through different mechanisms. ATV induces apoptosis and cell deaths in breast, prostate, and bladder cancer cells through autophagy (9, 21 and 22) and inducing chromosome breaks (23). ATV effectively induced apoptosis in ovarian cancer cells through activation of Jun N-terminal kinases (JNK) (24). ATV is able to induce oxidative stress in breast cancer cell results in apoptosis and cell deaths (25). Several mechanisms have been demonstrated for radiosensitizing effects of drugs such as AKT/mTOR signalling pathways and cox-2 inhibition (by celecoxib) (26), activated checkpoint kinase 2 (CHK2) ( by valproic acid) (27), ROS production (by mefenamic acid) (19), reduction of intracellular GSH content and HIF-1α expression (by oleanolic acid) (28), downregulated endogenous STAT1 expression (by zoledronic acid) (29), cell cycle G2/M arrest (by albendazole) (30), and autophagy inducing ( by vitamin D or vitamin D analogs) (31). Recently Jones *et al.* showed that anti-proliferative activity of ATV in the ovarian cancer cell lines was associated with induction of apoptosis, autophagy, cellular stress, and cell cycle G1 arrest via inhibition of AKT/mTOR and activation of the MAPK pathways. ATV inhibited cell adhesion and invasion, and finally ATV exhibited anti-metastasis (32). Metastasis is a challenge of cancer disease in patients, and ATV could diminish metastasis in patients with cancers. In our study, ATV alone exhibited anti-proliferation and apoptosis in breast and lung cancer cells, that is in accordance with previous studies. In this study, an additive and synergistic effect of ATV and IR were observed on the irradiated breast and lung cancer cells. Recently we showed that ATV protected testis and kidneys against toxicity induced by IR in mice. The antioxidant, anti-inflammatory, and anti-caspase-3 activities were main mechanisms of ATV in radioprotection (14, 15). 

The dual capabilities of ATV to act as an oxidant and radiosensitizing effect in breast and lung cancer cells, yet radioprotective effect in normal cells, supports the role of ATV as a promising agent for radiotherapy that could significantly improve the cancer treatment. 

## Conclusion

We demonstrated that the ATV sensitized human breast and lung cancer cells to radiation-induced apoptosis and death. Since, previously the radioprotective effects of ATV on normal cells and tissues were observed, this result provides a new indication of ATV for sensitizing the cancer cells to radiation therapy in the patients. 
